# Ischemia and No Obstructive Coronary Artery Disease: Prevalence and Correlates of Coronary Vasomotion Disorders

**DOI:** 10.1161/CIRCINTERVENTIONS.119.008126

**Published:** 2019-12-13

**Authors:** Thomas J. Ford, Eric Yii, Novalia Sidik, Richard Good, Paul Rocchiccioli, Margaret McEntegart, Stuart Watkins, Hany Eteiba, Aadil Shaukat, Mitchell Lindsay, Keith Robertson, Stuart Hood, Ross McGeoch, Robert McDade, Peter McCartney, David Corcoran, Damien Collison, Christopher Rush, Bethany Stanley, Alex McConnachie, Naveed Sattar, Rhian M. Touyz, Keith G. Oldroyd, Colin Berry

**Affiliations:** 1 Department of Interventional Cardiology, West of Scotland Heart and Lung Centre, Golden Jubilee National Hospital, United Kingdom (T.J.F., R.G., P.R., M.M., S.W., H.E., A.S., M.L., K.R., S.H., R.M., D. Collison., K.G.O., C.B.).; 2 British Heart Foundation Glasgow Cardiovascular Research Centre, Institute of Cardiovascular and Medical Sciences, University of Glasgow, United Kingdom (T.J.F., E.Y., N. Sidik., P.R., M.M., P.M., D. Collison, C.R., R.M.T., K.G.O., C.B.).; 3 Department of Interventional Cardiology, Gosford Hospital, New South Wales, Australia (T.J.F.).; 4 University of New South Wales, Sydney, Australia (T.J.F.).; 5 Department of Interventional Cardiology, University Hospital Hairmyres, East Kilbride, United Kingdom (R. McGeoch, N. Sattar).; 6 Robertson Centre for Biostatistics, Institute of Health and Wellbeing, University of Glasgow, United Kingdom (B.S., A.M.).

**Keywords:** angina pectoris, dyspnea, microvascular angina, prevalence, quality of life

## Abstract

Determine the prevalence and correlates of microvascular and vasospastic angina in patients with symptoms and signs of ischemia but no obstructive coronary artery disease (INOCA).

**Methods:**

Three hundred ninety-one patients with angina were enrolled at 2 regional centers over 12 months from November 2016 (NCT03193294). INOCA subjects (n=185; 47%) had more limiting dyspnea (New York Heart Association classification III/IV 54% versus 37%; odds ratio [OR], 2.0 [1.3–3.0]; *P*=0.001) and were more likely to be female (68% INOCA versus 38% in coronary artery disease; OR, 1.9 [1.5 to 2.5]; *P*<0.001) but with lower cardiovascular risk scores (ASSIGN score median 20% versus 24%; *P*=0.003). INOCA subjects had similar burden of angina (Seattle Angina Questionnaire) but reduced quality of life compared with coronary artery disease; subjects (EQ5D-5 L index 0.60 versus 0.65 units; *P*=0.041).

**Results:**

An interventional diagnostic procedure with reference invasive tests including coronary flow reserve, microvascular resistance, and vasomotor responses to intracoronary acetylcholine (vasospasm provocation) was performed in 151 INOCA subjects. Overall, 78 (52%) had isolated microvascular angina, 25 (17%) had isolated vasospastic angina, 31 (20%) had both, and 17 (11%) had noncardiac chest pain. Regression analysis showed inducible ischemia on treadmill testing (OR, 7.5 [95% CI, 1.7–33.0]; *P*=0.008) and typical angina (OR, 2.7 [1.1–6.6]; *P*=0.032) were independently associated with microvascular angina. Female sex tended to associate with a diagnosis of microvascular angina although this was not significant (OR, 2.7 [0.9–7.9]; *P*=0.063). Vasospastic angina was associated with smoking (OR, 9.5 [2.8–32.7]; *P*<0.001) and age (OR, 1.1 per year, [1.0–1.2]; *P*=0.032].

**Conclusions:**

Over three quarters of patients with INOCA have identifiable disorders of coronary vasomotion including microvascular and vasospastic angina. These patients have comparable angina burden but reduced quality of life compared to patients with obstructive coronary artery disease. Microvascular angina and vasospastic angina are distinct disorders that may coexist but differ in associated clinical characteristics, symptoms, and angina severity.

**Clinical Trial Registration:**

URL: https://www.clinicaltrials.gov. Unique identifier: NCT03193294.

## Abstract 2

Supplemental Digital Content is available in the text.

**WHAT IS KNOWN**

- Angina without obstructive coronary artery disease is common and may be due to underlying disorders including microvascular angina and vasospastic angina.
- These patients are at elevated risk for cardiovascular events (including acute coronary syndrome, repeated cardiovascular procedures, and heart failure hospitalization).

**WHAT THE STUDY ADDS**

- Over three quarters of patients with symptoms and signs of ischemia and no obstructive coronary artery disease have identifiable disorders of coronary vasomotion including microvascular and vasospastic angina.
- Traditional cardiovascular scores do not predict risk of coronary vasomotor disorders.
- Patients with ischemia and no obstructive coronary artery disease have similar angina burden but worse quality of life than obstructive coronary artery disease subjects.

Consensus guidelines for diagnosis and management of stable ischemic heart disease are predominantly shaped by the burden of epicardial disease.^1,2^ Therefore, patients with symptoms and signs of ischemia and no obstructive coronary artery disease (INOCA) pose a challenge to treating physicians in both identification and manage.^3^ Microvascular angina (MVA) and vasospastic angina (VSA) are relevant causes that are rarely identified during coronary angiography.^3–5^ The reference diagnostic approach involves direct assessment of coronary vascular function typically using pharmacological probes.^1–9^

Wider developments in invasive coronary physiology coupled with better awareness of coronary vasomotion disorders have led to recently standardized diagnostic criteria.^8,10–12^ In the CorMicA study, we showed that an interventional diagnostic procedure (IDP: measurement of coronary flow reserve [CFR], microcirculatory resistance [IMR], and vasomotor responses to intracoronary acetylcholine [ACh]) helped identify the cause of angina with tailored treatment improving symptoms and quality of life compared with standard care.^7^

In this prespecified CorMicA analysis, we analyzed the overall prevalence and predictors of MVA and VSA using a prospective all-comer study design incorporating reference invasive tests of coronary vascular function in patients with angina.

## Methods

### Study Design and Participants

We screened elective adult referrals to 2 regional hospitals (Golden Jubilee National Hospital and University Hospital Hairmyres) providing invasive cardiac services to the West of Scotland (population 2.5 million). This all-comer design involved INOCA subjects being offered invasive testing as part of a prespecified analysis (CorMicA).^13^ The invasive protocol is summarized in the Data Supplement (Appendix). Outpatients undergoing clinically indicated, elective diagnostic coronary angiography for the investigation of angina were screened on the day of the procedure using the Rose angina questionnaire.^14^^R14^ Patients who provided a response consistent with definite or probable angina were invited to participate.^14^ Exclusion criteria included a noncoronary indication for invasive angiography, for example, valve disease, cardiomyopathy, and inability to give informed consent. The ASSIGN Score is a validated cardiovascular risk score providing estimated 10-year risk of cardiovascular events incorporating social deprivation and family history of cardiovascular disease.^15^ The West of Scotland Research Ethics Committee approved the study (reference 16/WS/0192) and all subjects gave informed consent. The data that support the findings of this study are available from the corresponding author on reasonable request.

### Interventional Diagnostic Procedure

Coronary thermodilution was used to measure CFR (abnormal <2.0,^16^ the index of microcirculatory resistance [IMR; abnormal ≥25]^17^ and fractional flow reserve, abnormal ≤0.80)^18^ during intravenous infusion of adenosine (140 µg/kg per minute). ACh infusions were followed by provocation testing for inducing epicardial spasm using 100 mcg bolus of ACh over 20 seconds (Figure I in the Data Supplement).

### Definitions

- INOCA: syndrome of patients with either symptoms and/or signs of ischemia but found to have no obstructive coronary artery disease (CAD).^3^
  - All subjects must have either definite (typical) or probable (atypical) angina on Rose questionnaire.^14^
- Obstructive CAD: ≥50% diameter stenosis and fractional flow reserve ≤0.80 during coronary angiography.
- VSA^12^: reproduction of angina symptoms, ischemic ECG changes with ≥ 90% constriction in major epicardial artery.
- MVA^8^: angina, no obstructive CAD plus objective evidence of coronary microvascular dysfunction (as defined by abnormal response to intracoronary ACh and/or systemic adenosine [CFR and/or IMR])
  - CFR<2
  - IMR≥25
  - ACh response (microvascular spasm) defined by reproduction of angina, ST segment deviation (≥1 mm) and absence of significant epicardial coronary vasoconstriction during ACh (<90% epicardial constriction)
- Mixed microvascular and VSA: angina with no obstructive CAD plus BOTH evidence of invasive coronary microvascular dysfunction and epicardial vasospasm to ACh (≥90% epicardial constriction)
- Noncardiac chest pain: normal coronary vascular function during IDP assessment
- Myocardial bridging^19^: angiographic evidence of discrete systolic compression (milking effect ≥10% systolic compression during the cardiac cycle)
- Endothelial dysfunction is defined by ≥20% luminal constriction during ACh infusion (up to 10^−^^4^ M).^20^

### Statistical Methods

Statistical analyses were performed with Prism 7.0 (GraphPad, La Jolla, CA) and SPSS 25.0 (SPSS, Chicago, IL). Categorical variables are expressed as number and percentage of patients. Continuous variables are presented as means (SD) or median (IQR). Differences between groups were assessed using 1-way ANOVA, Mann-Whitney *U* tests, Chi squared, or Fisher exact tests as appropriate. Prespecified subgroup analysis of MVA and VSA groups was performed using unpaired t test. *P* value of ≤0.05 was considered statistically significant.

Binary logistic regression with MVA and VSA as outcomes was performed using prespecified variables of interest: sex, age, symptom characteristics, results from noninvasive exercise tolerance testing, cardiovascular risk factors (hypertension, diabetes, dyslipidemia, smoking, previous myocardial infarction), chronic pain disorders, and family history. Receiver operator curve were created for the models to see whether the variables could predict MVA or VSA as shown in Figure II in the Data Supplement. To assess discrimination of the regression model, we used the Harrell’s c-statistic corresponding to the area under the receiver operating characteristic curve to assess the goodness of fit and diagnostic accuracy.

Angina and quality of life assessment is detailed in the Data Supplement (Appendix).

## Results

### Angina: Prevalence of Obstructive CAD and INOCA

**Figure 1.**
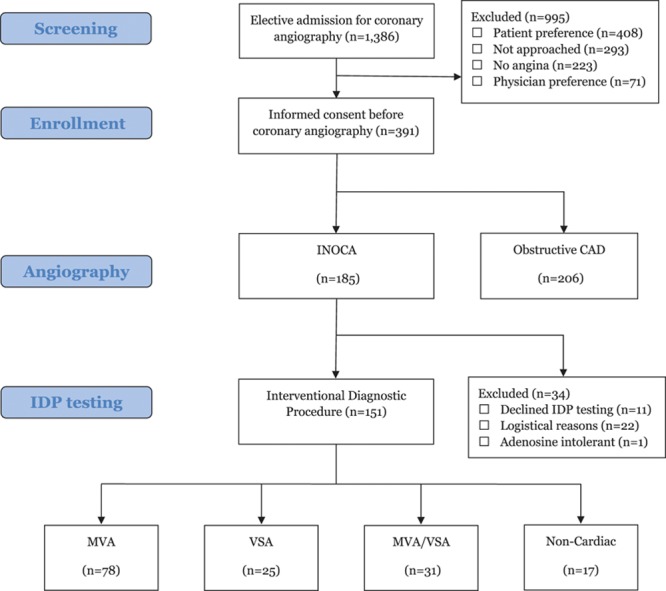
**Study overview—prevalence of coronary vasomotion disorders in ischemia and no obstructive coronary artery disease (INOCA**). Figure showing screening and enrollment process with a total of 185 INOCA patients and 206 oCAD patients. IDP indicates interventional diagnostic procedure; MVA, microvascular angina; oCAD, obstructive epicardial disease; and VSA, vasospastic angina.

Between November 25, 2016 and December 11, 2017, three hundred ninety-one patients with probable or definite angina were enrolled from 1386 elective referrals immediately before invasive coronary angiography. The majority of patients who were not enrolled lacked eligibility criteria (Figure 1). Coronary angiography revealed obstructive CAD and no obstructive CAD in 206 (53%) and 185 (47%) subjects, respectively.

### Demographics and Quality of Life Between CAD and INOCA

Subjects with obstructive epicardial disease (oCAD) tended to be older and have a higher prevalence of previous cardiac events and dyslipidemia (Table II in the Data Supplement). INOCA subjects were more likely to be female (68% versus 38% in oCAD; OR, 1.9 [1.5–2.5]; *P*<0.001) and have a higher incidence of chronic pain disorders at baseline (23% versus 10%; *P*<0.001). The INOCA population were around 60% more likely to have undergone a previous invasive coronary angiogram (32% in INOCA versus 22% in oCAD; OR, 1.58 [1.01–2.48]; *P*=0.046).

Approximately two thirds of subjects in both groups underwent symptom-limited exercise treadmill testing as part of physician-reported standard care work-up. The presence of an abnormal exercise treadmill testing with inducible ischemia was higher in the oCAD group (81% versus 47%; *P*<0.001). Typical angina was more prevalent in the oCAD group (85% versus 64%; P<0.001) whereas the INOCA subjects had more limiting dyspnea (New York Heart Association classification III/IV 54% versus 37%; OR, 2.0 [1.3–3.0]; *P*=0.001). The angina severity scores showed a similar overall angina burden (SAQ summary score, 53.6 in oCAD versus 51.3 in INOCA; *P*=0.224). At baseline, most of the participants had daily or weekly angina (Seattle Angina Questionnaire, frequency score≈60), with mild to moderate angina limitation worse in the INOCA group (mean Seattle Angina Questionnaire, limitation 52.5 versus 58.3; *P*=0.018). Quality of life at baseline assessed by EQ5D-5 L index was significantly lower in the INOCA group (0.60 versus 0.65; *P*=0.041).

### INOCA: Prevalence of Vasomotion Disorders

**Figure 2.**
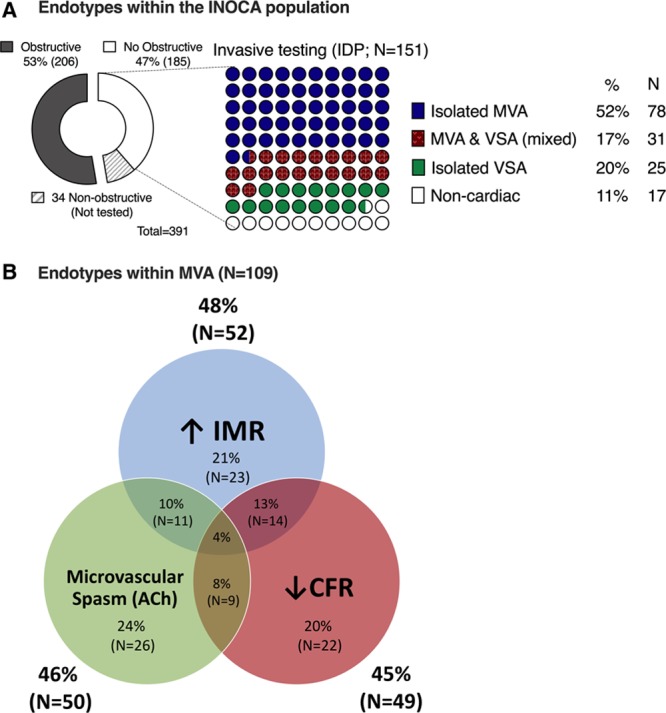
**Invasive assessment for coronary vasomotion disorders in angina**. **A**, Shows different diagnoses within ischemia and no obstructive coronary artery disease (INOCA) population while (**B**) shows the heterogenous nature of microvascular angina. ACh indicates acetylcholine; CFR, coronary flow reserve; IDP, interventional diagnostic procedure; IMR, index of microcirculatory resistance (abnormal ≥25); MVA, microvascular angina; and VSA, vasospastic angina.

Coronary vascular function was immediately assessed after diagnostic angiography in 151 of 185 INOCA participants (Table 1). Thirty-four (18%) subjects did not have coronary function assessed because of logistical reasons or because of patient and clinician preferences. The mean age of subjects who underwent the IDP was 60.9 (±10.0) years, and three quarters were female (n=111 [74%]). Figure 2A shows the different diagnoses within the INOCA population, Figure 2B shows the heterogenous nature of MVA with various types of CMD including both structural and functional disorders. Myocardial bridging was noted in 22 (15%) subjects; however, we did not perform dobutamine challenge to assess the ischemic potential of the tunnelled segment. Approximately one-third of the study population who underwent an IDP (34%,N=51) had a previous invasive coronary angiogram with no obstructive CAD (range, 1–5 previous angiograms).

**Table 1.**
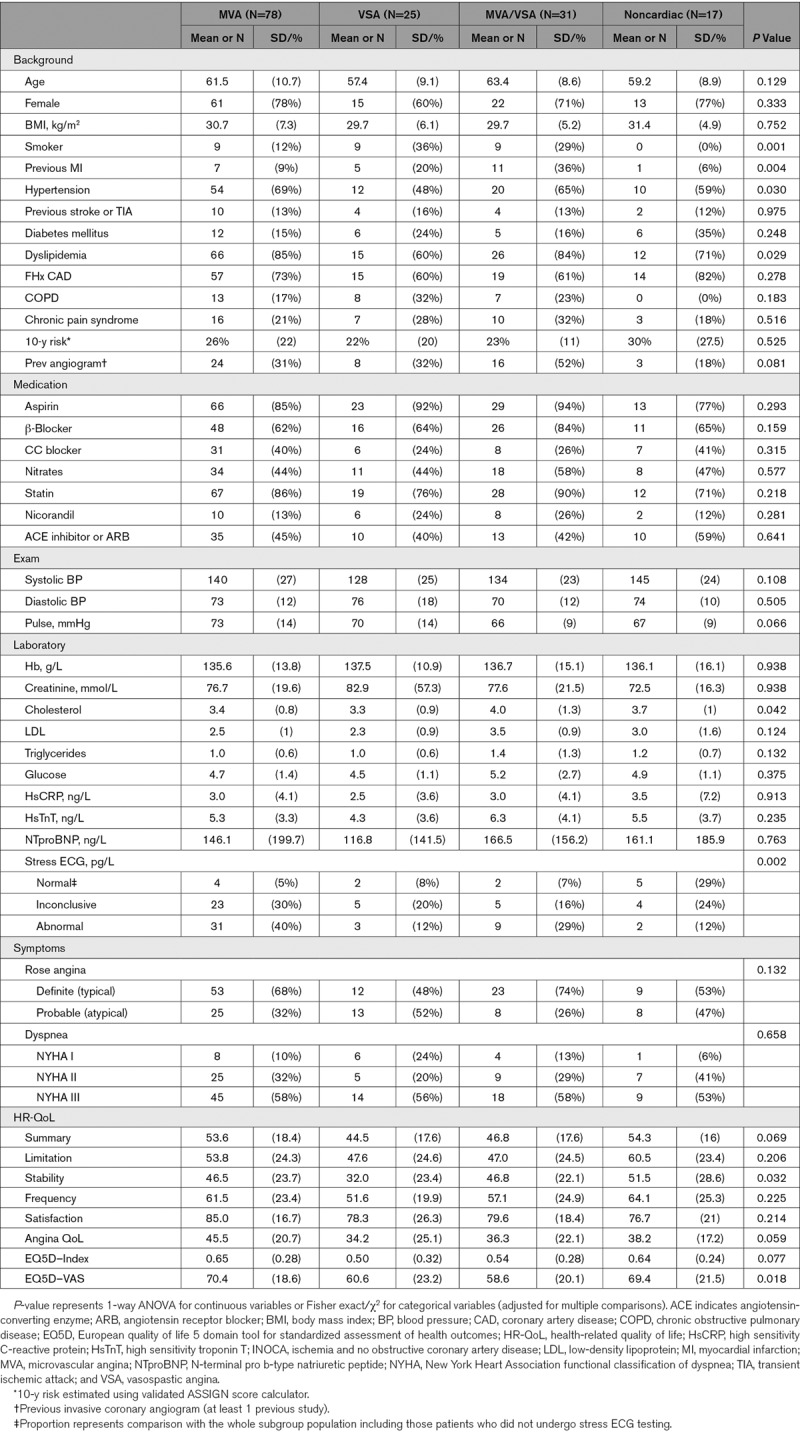
Patient Demographics According to INOCA Endotype

The procedural characteristics are shown in Table 2. The target artery was the left anterior descending in 132 subjects (87%) with no serious adverse events occurring related to the IDP (Table I in the Data Supplement). Across the INOCA cohort, the mean fractional flow reserve was 0.88 (±0.06). Epicardial disease severity assessed using the Gensini score and fractional flow reserve were similar between the groups. Angiographically normal coronary arteries (no demonstrable luminal irregularities) were significantly more prevalent in the noncardiac cohort compared with the subjects with mixed MVA/VSA (OR, 6.53 [1.41–30.3]; *P*=0.016).

**Table 2.**
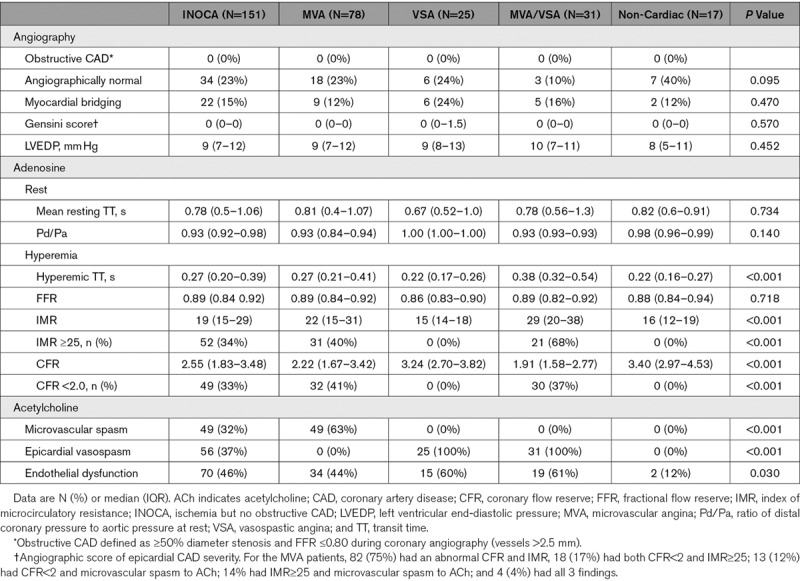
Procedural Characteristics: Invasive Evaluation of INOCA Subjects

### Multivariate Associates of MVA

In a logistic regression model with MVA as the outcome, traditional cardiovascular risk factors including risk assessed using the ASSIGN score were not associated with MVA (Table III in the Data Supplement). Ischemia on exercise treadmill testing was associated with MVA (OR, 7.5 [95% CI, 1.7–33.0]; *P*=0.008) and typical angina was also predictive (OR, 2.7 [95% CI, 1.1–6.6]; *P*=0.032). Female sex (OR, 2.7 [0.9–7.9]; *P*=0.063) and increasing age (OR, 1.1 per year [1.0–1.2]; *P*=0.051) were positively associated with MVA, although the relationships were not statistically significant. The fitted regression model for MVA showed excellent discrimination (*c*-statistic, 0.85 [95% CI, 0.78–0.92]; *P*<0.001; Figure II in the Data Supplement).

### Multivariate Associates of VSA

In a logistic regression model with VSA as the outcome, age, and cigarette smoking were the only cardiovascular risk factors positively associated with the diagnosis (Table III in the Data Supplement). For every year increase in age, the odds of VSA grew by 10% (OR, 1.1 [95% CI, 1.0–1.2]; *P*=0.032). Higher traditional cardiovascular risk score (ASSIGN) was associated with a reduced risk of VSA (OR, 0.9 [0.9–1.0]; *P*=0.013). Inducible ischemia on exercise tolerance testing was associated with a reduced risk of VSA (OR, 0.3 [95% CI, 0.1–0.9]; *P*=0.040); however, angina characteristics (eg, atypical angina) were not associated with VSA diagnosis. The fitted regression model for VSA showed excellent discrimination (*c*-statistic, 0.82 [95% CI, 0.74–0.89]; *P*<0.001).

### MVA and VSA: Quality of Life Differences

**Figure 3.**
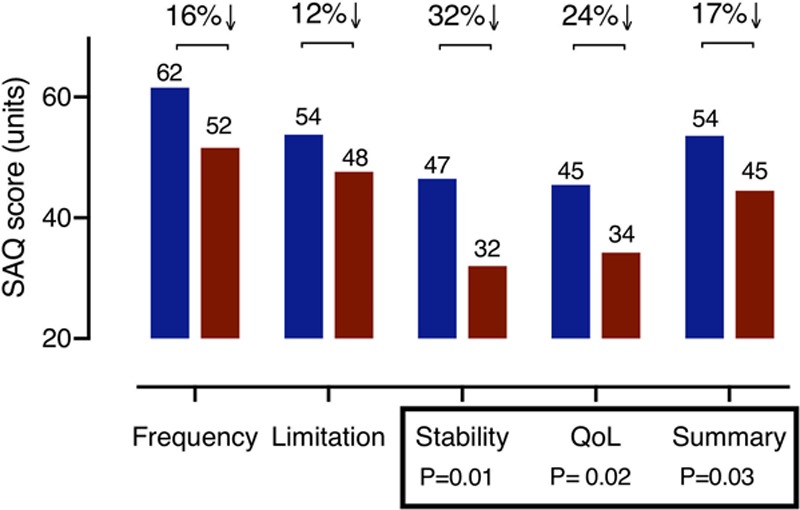
**Differences in angina and quality of life between microvascular angina (MVA) and vasospastic angina (VSA**). Figure showing the differences in angina and quality of life (QoL) score with VSA group having almost 20% lower overall angina summary score compared with MVA. SAQ indicates Seattle Angina Questionnaire.

The VSA group had almost 20% lower overall angina summary score compared with MVA (Figure 3; mean 45 versus 54 units; −9 [95% CI, −17 to −1]; *P*=0.028). VSA subjects had over 30% worse angina stability (mean 32 versus 47 units; −15 [95% CI, −25 to −4]; *P*=0.009]) with 24% lower angina-related quality of life (mean 34 versus 45 units; −11 [95% CI, −21 to −1]; *P*=0.024). There were trends toward greater angina frequency (mean 52 versus 62 units; −10 units [95% CI, −21 to 1]; *P*=0.067); however, this was not statistically significant (Figure 3; lower score indicates more severe angina). The EQ-5D-5L index revealed a 25% lower overall quality of life in the VSA group compared with MVA (mean, 0.5 versus 0.65; −0.15 units [95% CI, −0.28 to −0.02]; *P*=0.023). Notably, the group with mixed MVA/VSA had similar angina characteristics but 18% worse overall quality of life score compared with the group with isolated MVA (EQ5D difference −12 units, −20 to −4; *P*=0.006).

There were notable differences in baseline characteristics including smoking, dyslipidemia, and inducible ischemia on exercise treadmill testing. The mean age of VSA tended to be younger (−4.0 years [95% CI, −8.5 to 0.47]; *P*=0.079) and systolic blood pressure lower compared with MVA (−12 mmHg; −23 to 0 mmHg; *P*=0.051).

## Discussion

In this prospective analysis of coronary vasomotion disorder prevalence, we used reference invasive diagnostic tests in an undifferentiated population of patients with angina undergoing clinically indicated coronary angiography. The main findings were: (1) around one half of patients with angina had symptoms and/or signs of ischemia but no obstructive CAD (INOCA); (2) disorders of coronary vasomotion are common in INOCA with over three quarters having a disorder of coronary vascular function and symptom burden comparable to obstructive CAD; (3) MVA and VSA may coexist but differ in risk factor profiles, noninvasive investigations, angina symptoms and invasive coronary physiology.

### Building on Prior Studies

The WISE (Women’s Ischemia Syndrome Evaluation) study evaluated baseline CFR invasively in 189 women with angina and no obstructive coronary arteries and identified 74 (39%) patients with coronary microvascular dysfunction (defined as CFR≤2.32). The protocol did not involve universal administration of ACh during coronary reactivity testing.^9^ Aziz et al^21^ reported the largest case series of patients with INOCA in Europe (n=1379), with intracoronary ACh testing (without adenosine) disclosing a high prevalence of vasomotor disorders including 26% with epicardial spasm and 33% with microvascular spasm, similar to our population. Sara et al reported a retrospective analysis of 1552 patients with INOCA in whom 64% had underlying CMD. Their protocol involved intracoronary Doppler measurements during simultaneous infusion of ACh and then adenosine to assess endothelium-dependent and endothelium-independent function, respectively. The protocol did not involve provocation testing for coronary vasospasm.^22^ Existing studies of disease prevalence may be prone to selection bias known as referral filter bias, by enrolling patients referred to tertiary care centers with a special interest in this problem.^23,24^ Lee et al described a cohort of 139 patients who had a diagnosis of INOCA based on invasive physiological testing combined with intravascular ultrasound. ACh was administered into the LAD to assess endothelial function, although the conventional high dose bolus of ACh (100 mcg over 20 seconds) was given at a modified rate over 3 minutes. The short plasma half-life of ACh would render the intracoronary concentration significantly lower in their study, which may explain why no cases of epicardial vasospasm were identified. The Japanese established ACh testing for VSA and the 2014 Japanese Cardiovascular Society guidelines on the invasive diagnosis of vasospasm using ACh established an intracoronary bolus dose of 20 to 100 μg administered over 20 seconds.^25^ The higher rate of spasm in our study aligns with other published cohorts,^26^ and it is plausible that no subjects in their cohort were diagnosed with VSA due to the different ACh protocol. Interestingly, Lee’s cohort had similar levels of endothelial impairment as CorMicA subjects (44% versus 46%); nevertheless, microvascular function assessed with adenosine (CFR/IMR) was quite different. Abnormal vasodilator capacity (low CFR) was noted in only 7% versus 33% in CorMicA and increased microvascular resistance (high IMR) was noted in only 21% versus 34% in our study. There are clear differences in the referral pathways and population studied that could explain these differences with CorMicA patients being nearly 10 years older on average, with more obesity, dyslipidemia, and greater cardiac risk. The enrollment period of 5-years implies that the population (n=139 participants) was preselected. These studies have provided unique insights into the pathophysiology of INOCA but by design each had limitations raising uncertainty about the prevalence and significance of vasomotor disorders. Our study had a consecutive approach to enrollment during usual care and 151 subjects were included within 12 months.

In addition to showing that the majority of INOCA subjects have demonstrable abnormalities in coronary vascular function, we showed they had more limiting dyspnea compared with oCAD subjects (New York Heart Association classification III/IV 54% versus 37%; OR, 2.0 [1.3–3.0]; *P*=0.001). This is important given the emerging evidence that coronary microvascular dysfunction in INOCA subjects is closely linked with the syndrome of heart failure with preserved ejection fraction.^27^

Patel et al^28^ presented US data of 398 978 patients referred for coronary angiography, demonstrating that >60% had no obstructive CAD and 39% of patients had angiographically normal coronary arteries. More recent data are varied but the prevalence of no obstructive CAD was still almost 60% in the same groups most recent analysis of the national US cath PCI registry (almost 700 000 elective coronary angiograms).^29^ The Ischemia trial had to exclude over 20% of patients with angina owing to no obstructive CAD on CT coronary angiography despite moderate to severe ischemia on functional assessment.^30^ Other centers in the United Kingdom report at least 1/3 of patients with angina undergoing invasive coronary angiography have no obstructive CAD.^31^

### MVA and VSA: Distinct Coronary Vasomotion Disorders

MVA and VSA may coexist,^32^ and 1/5 of the participants in our cohort had both disorders. Nevertheless, prespecified analysis of isolated MVA and VSA subgroups revealed important differences with implications for investigation and management of patients with INOCA. First, MVA and VSA subjects differed in risk factor profiles and associated characteristics. Traditional cardiovascular risk scores do not discriminate whether subjects have MVA. This has previously been highlighted by the iPower investigators.^33^ VSA was independently associated with older age and smoking but was interestingly inversely associated with traditional risk assessed by the ASSIGN score. VSA subjects were 3× as likely to have suffered a previous myocardial infarction but with lower prevalence of dyslipidemia compared with the patients with MVA. This is critical information which supports a different phenotype and underlying pathophysiology and supports identification with tailored therapies. This is evidenced by near identical prescribed therapies at baseline with 2/3 of both patients with MVA and VSA on β-blockers despite them being relatively contraindicated in VSA.^2^ The burden of medications for most subjects was substantial.

Interestingly, serum biomarker levels including inflammatory markers, troponin, and BNP (B-natriuretic peptide) were similar between the INOCA groups. There are a few explanations for this but coronary vasomotor disorders including coronary microvascular dysfunction have only weak correlation with these biomarkers.^34^ Other nontraditional risk factors may be more important in functional disorders. Furthermore, most of the subgroups were on established treatment before baseline bloods, which could confound and normalize the biomarkers.

### Strengths and Limitations

Our study has several strengths. First, enrollment across 2 regional centers providing care to a large population (2.5 million) over a wide geographic area (half of Scotland), with a prospective, all-comer approach to enrollment of a comparatively large population increases the generalizability of our findings. Second, participants underwent invasive characterization of coronary vasomotion using established diagnostic criteria with a comprehensive protocol aligned with international standards.^8,12^ The diagnostic accuracy of intracoronary ACh for diagnosing coronary spasm during invasive coronary angiography alone is excellent (sensitivity, 90%; specificity, 99%).^35^ Third, this multimodality protocol provided novel insights into disease mechanisms and clinical correlates, identifying distinct endotypes within an otherwise undifferentiated population of patients. We designed the IDP to be performed on a single major epicardial artery (typically the LAD for pragmatic reasons); however, regional variations in microvascular function and propensity to epicardial coronary spasm are well recognised.^36,37^ Furthermore, it was not possible to recruit all patients undergoing invasive angiography resulting in the potential for bias. Vasomotion disorder prevalence may vary according to local referral practices and multinational studies are warranted.

Our findings have wider implications, including the accuracy of noninvasive tests used for patients with stable chest pain in the clinic. Functional tests are recommended for diagnosing MVA^2,8^; however, anatomic testing with CT coronary angiography may result in false reassurance for patients with no obstructive CAD but underlying MVA and VSA. These patients are predominantly women.^38^ Discontinuation of therapy by protocol in patients with undiagnosed MVA may be one explanation for why CT coronary angiography-guided management was associated with more angina and poorer quality of life during longer term follow-up in the SCOT-HEART trial (Scottish Computed Tomography of the HEART Trial).^39^ The prevalence and clinical significance of vasomotor disorders in patients with angina classified with no obstructive CAD by CT coronary angiography is being prospectively assessed in the CorCTCA (Coronary Microvascular Function and CT Coronary Angiography) study.

## Conclusions

Around one half of suspected patients with angina undergoing invasive coronary angiography have symptoms and signs of INOCA. These patients report similar angina burden with worse quality of life than obstructive CAD subjects. The majority of INOCA subjects have underlying disorders of coronary vasomotion (microvascular and VSA). Identification of these common disorders may facilitate patient education and distinct medical treatments.

## Acknowledgments

We gratefully acknowledge the patients, staff, and British Heart Foundation who supported this study.

## Sources of Funding

This research was funded by the British Heart Foundation (PG/17/2532884;RE/13/5/30177; RE/18/6134217).

## Disclosures

Dr Berry is employed by the University of Glasgow which holds consultancy and research agreements with companies that have commercial interests in the diagnosis and treatment of angina. The companies include Abbott Vascular, AstraZeneca, Boehringer Ingelheim, GSK, HeartFlow, Menarini Pharmaceuticals, Opsens, Philips, and Siemens Healthcare. Dr Oldroyd has received consultant and speaker fees from Abbott Vascular and Boston Scientific. Dr Watkins has received consultant and speaker fees from Boston Scientific. Dr Rocchiccioli has received consultant and speaker fees from Astra Zeneca. Dr Robertson has received educational support from Abbott Vascular and speaker fees from Astra Zeneca. None of these companies have had any involvement with this study. Dr McEntegart has a proctoring agreement with Boston Scientific and Vascular Perspectives. The other authors report no conflicts.

## Supplementary Material
