# Supplementary material for: Ischemia and No Obstructive Coronary Artery Disease: Prevalence and Correlates of Coronary Vasomotion Disorders
Source: Circ Cardiovasc Interv. 2019 Dec 13;12(12):e008126. doi: 10.1161/CIRCINTERVENTIONS.119.008126 (PMC6924940; doi:10.1161/CIRCINTERVENTIONS.119.008126)
Supplement: Supplementary file 1 [file hcv-12-e008126-s001.pdf]

Supplemental Material

This appendix has been provided by the authors to give readers additional information about the prevalence and correlates of coronary vasomotion disorders paper.

Table of Contents

1. ***Interventional Diagnostic Procedure (IDP) and Angiographic Analysis 3***

2. ***Quantitative Coronary Angiography (QCA) 4***

3. ***Angina and Quality of Life Assessments 5***

4. ***Statistical Methods……………………………………………………………………………………6-7***

5. ***Figure 1 – Cardiac catheterisation laboratory protocol…………………………………….8***

6. ***Figure 2 - ROC curves for identification of MVA & VSA 9***

7. ***Supplemental Table 1 - Adverse events during acetylcholine testing 10***

8. ***Supplemental Table 2 – Patient demographics and health related quality of life: comparison of obstructive CAD and INOCA…………………………………………………11-12***

9. ***Supplemental Table 3 - Multivariate associates of MVA and VSA in the INOCA population (n=151)…………………………………………………………………………………………….13***

10. ***References...144***

1. Interventional Diagnostic Procedure (IDP) and Angiographic Analysis

We measured the myocardial fractional flow reserve (FFR; abnormal ≤0.80) to assess for flow-limiting CAD.[1] Coronary flow reserve (CFR; abnormal <2.0) and the index of microcirculatory resistance (IMR; abnormal >25) reflected coronary vasodilator reserve and microvascular resistance, respectively.[2, 3] These parameters were derived in real-time by coronary thermodilution at rest and during hyperaemia using automated software, as previously described. An intravenous infusion of adenosine (140 µg·kg−1·min−1) was administered via a large peripheral vein to induce steady-state maximal hyperaemia. Thermodilution was performed by manual intra-coronary injection of 3 ml of normal saline (room temperature) via the guiding catheter with the diagnostic guidewire in situ in the target coronary artery. The pressure- and temperature sensor on this wire was positioned at least 6 – 9 cm distally in the lumen of the coronary artery. CFR was calculated using thermodilution as resting mean transit time divided by hyperaemic mean transit time. CFR takes account of vasodilatation of the conduit coronary artery and its microvasculature. IMR was calculated as the product of the mean distal coronary artery pressure and the mean transit time measured simulataneously during hyperaemia. An increased IMR (≥25) reflects coronary microvascular dysfunction. Fractional flow reserve (FFR) represents the ratio of the mean distal coronary pressure to mean aortic pressure during maximal hyperaemia (abnormal FFR ≤0.80).

Coronary Vasoreactivity Testing

We assessd endothelium-dependent coronary vasomotor function using sequential intra-coronary infusions of incremental doses of acetylcholine (ACh) via the guiding catheter.

The infused doses of ACh were 0.182, 1.82, and 18.2 µg/mL (10-6, 10-5, and 10-4 mol/L, respectively) at a flow rate of 1 ml/minute for 2 minute periods via a mechanical infusion pump as shown in supplemental Figure 1. We then performed provocation testing for epicardial coronary artery spasm using a 100 µg bolus of ACh (5.5 ml of 10-4 mol/L over 20 seconds – reduced to 50 µg for the right coronary artery to mitigate bradycardia), and finally non-endothelial vasodilator function was assessed by administration of 300 µg of glyceryl trinitrate in a 3-mL bolus. Adverse events during acetylcholine testing was shown in supplementalTable 1.

Invasive coronary angiography was performed via the radial artery in line with standard care at the participating hospitals and adjunctive coronary function tests were performed in patients with no obstructive CAD. We allowed glyceryl trinitrate (GTN) as a radial cocktail but not calcium channel antagonists. The half-life of GTN is around two minutes and thus after 10 minutes, only 3% of the medication is active and is unlikely to affect resting physiology nor mask a positive vasoreactivity test using ACh.[4] The IDP was focused on a single major coronary artery, usually the left anterior descending (LAD) artery. If technical factors precluded assessment of the LAD e.g. tortuous anatomy, then the left circumflex or right coronary artery was selected.

2. Quantitative Coronary Angiography (QCA)

Quantitative coronary analysis of the target coronary artery was performed using computer-assisted angiographic analysis (QAngio XA7.3, Medis, Leiden, Netherlands) by a trained cardiologist. Fluoroscopic images from two angles at least 30° apart were

acquired. The coronary artery (typically left anterior descending artery) measurements were performed in the region where the greatest change had occurred during coronary reactivity testing.[5] End-diastolic cine frames that best show the segment were selected, and calibration of the video and cine images was performed. Coronary artery diameter change (% from baseline) was measured in response to both ACh and glyceryl trinitrate. Severe endothelial dysfunction was defined by ≥20% luminal constriction during ACh infusion (up to 10−4M); this finding implies significant reduction in coronary artery blood flow with prognostic implications when compared with patients whose arteries were <20% constricted.[6] A second trained observer (PM) performed QCA on a consecutive sample of 10% of cases, with high concordance for measurements of percentage lumen diameter vasoconstriction during ACh vasospasm assessment (intraclass correlation coefficient for average measures 0.96; 95% CI 0.88-0.99; p<0.001) and Gensini angiographic score (intraclass correlation coefficient for average measures 0.99; 95% CI 0.96-1.00; p<0.001).

3. Angina and Quality of Life Assessments

The Rose-Angina questionnaire was administered on the day of the angiogram and only patients with definite or possible angina were eligible to participate.[7] The Seattle Angina Questionnaire (SAQ) is a self-administered, disease-specific measure of angina severity that is valid, reproducible and sensitive to change.[7] The SAQ quantifies patients' physical limitations caused by angina, the frequency of and recent changes in their symptoms, their satisfaction with treatment, and the degree to which they perceive their disease to affect their quality of life. Each scale is transformed to a score of 0 to 100, where higher

scores indicate better function (e.g. less physical limitation, less angina, and better quality of life).[8] The summary score (SAQSS) averages the domains of angina limitation, frequency and quality of life to provide an overall metric of angina severity.[9] Quality of life was assessed using validated, self-administered questionnaire (EuroQOL EQ-5D-5L).[10] This is a widely used standardized instrument for measuring generic health status whereby higher scores represent better quality of life (from −0.59 – 1.00 scale).

4. Statistical Methods

Statistical analyses were performed with Prism 7.0 (GraphPad, La Jolla, CA) and SPSS 25.0 (SPSS, Chicago, IL). Categorical variables are expressed as number and percentage of patients. Continuous variables are presented as means (SD) or median (IQR). Differences between groups were assessed using one-way ANOVA, Mann-Whitney tests, Chi squared or Fisher's exact tests as appropriate. Prespecified subgroup analysis of MVA & VSA groups was performed using unpaired t-test. P-value of ≤ 0.05 was considered statistically significant.

Binary logistic regression with MVA and VSA as outcomes was performed using prespecified variables of interest: sex, age, symptom characteristics, results from non-invasive exercise tolerance testing (ETT), cardiovascular risk factors (hypertension, diabetes, dyslipidemia, smoking, previous MI), chronic pain disorders and family history. Receiver operator curve (ROC) were created for the models to see whether the variables could predict MVA or VSA as shown in supplemental Figure 2. To assess discrimination of the regression model, we used the Harrell's c-statistic corresponding to the area under

the receiver operating characteristic curve (AUC - ROC) to assess the goodness of fit and diagnostic accuracy.

5. Supplemental Figure 1 – Cardiac catheterisation laboratory protocol

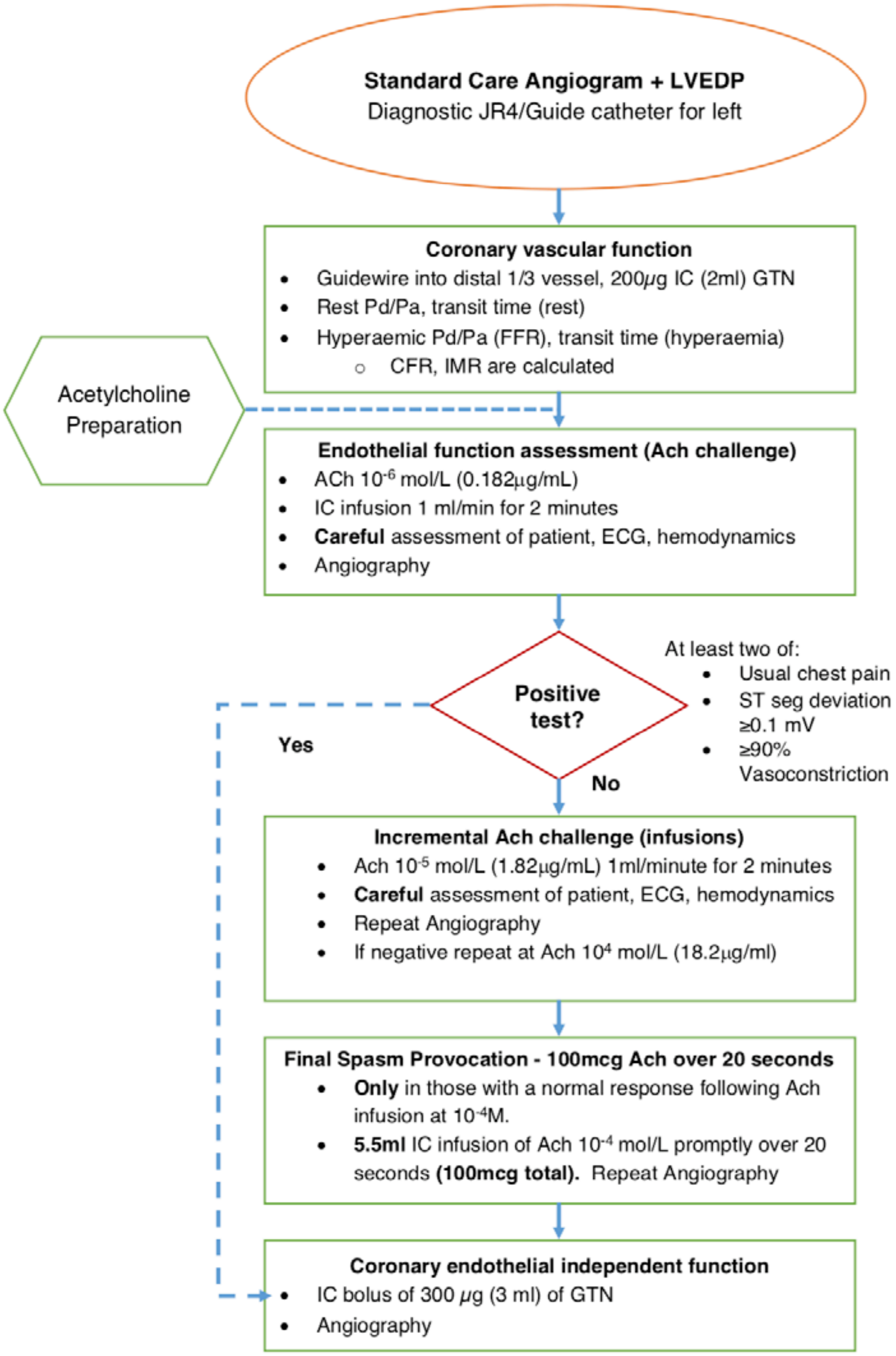

ACh – acetylcholine, ECG – 12 – lead electrocardiography, FFR – fractional flow reserve, GTN – glyceryl trinitrate, IC – intracoronary, JR4 – Judkins right shaped coronary catheter, LVEDP – left ventricular end-diastolic pressure, ST – ST ECG segment.

6. Supplemental Figure 2 - ROC curves for identification of MVA & VSA

Receiver operating characteristics (ROC) curves with area under the ROC curve (AUC) values of 0.85 for MVA (N=109; P<0.001) and 0.82 for VSA (N=56; P<0.001).

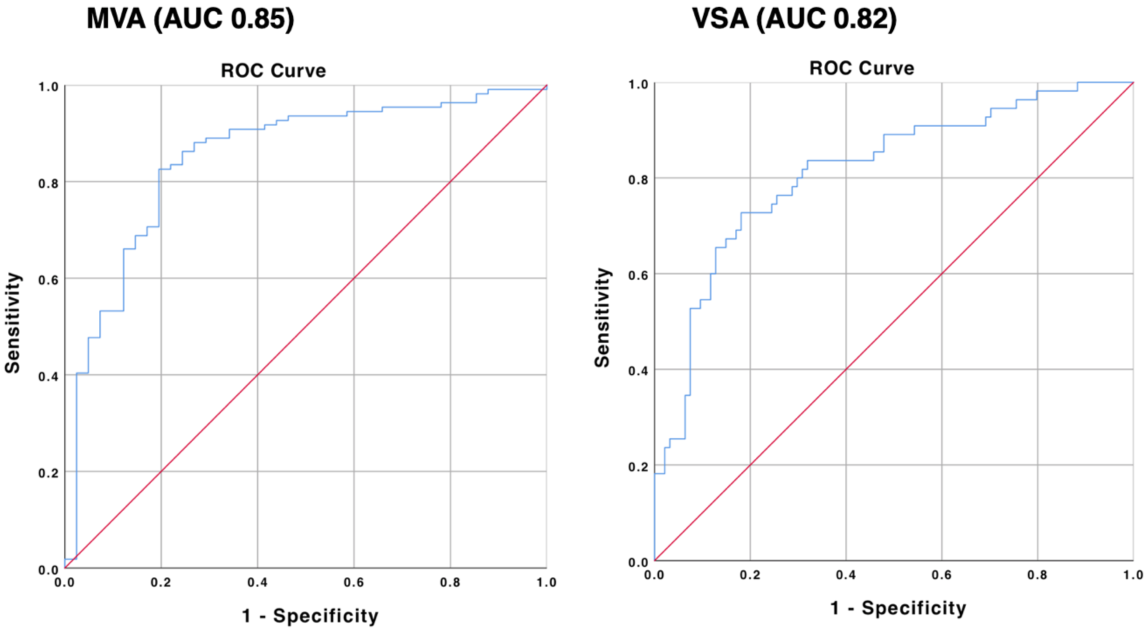

7. Supplemental Table 1 - Adverse events during acetylcholine testing

		All Patients
AF during Ach provocation test	N (n missing)	151 (0)
	Yes	9 (6.0%)
	No	142 (94.0%)
Self-limiting pause during Ach provocation test	N (n missing)	150 (1)
	Yes	49 (32.7%)
	No	101 (67.3%)
Bradycardia (necessitating termination of study protocol)	N (n missing)	150 (1)
	Yes	5 (3.3%)
	No	145 (96.7%)

Self-limiting pauses with propensity to transient atrial arrhythmias were common physiological effects of acetylcholine that were recorded but not considered detrimentral if requiring no additional treatment.

8. Supplemental Table 2 – Patient demographics and health related quality of life: comparison of obstructive CAD and INOCA.

		Obstructive CAD (N=206)		INOCA (N=185)		P-value
		Mean or	SD or	Mean or	SD or	
Baseline Clinical	Age	**63.0**	(9.6)	**61.3**	10.0	0.081
	Female	**79**	(38%)	**126**	(68%)	<0.001
	Body mass index (kg/m^2)	**29.3**	(4.9)	**30.2**	6.4	0.117
	Current Smoker	**50**	(24%)	**38**	(21%)	0.374
	Previous myocardial infarction	**59**	(29%)	**30**	(16%)	0.008
	Previous stroke or TIA	**15**	(7%)	**23**	(12%)	0.127
	Diabetes Mellitus	**53**	(21%)	**39**	(21%)	0.345
	Dyslipidemia	**181**	(88%)	**147**	(79%)	0.028
	Family History of CVD	**149**	(72%)	**124**	(67%)	0.271
	Predicted 10-year CHD risk*	**24%**	(17,39)	**20%**	(11,33)	0.003
	Chronic obstructive pulmonary	**35**	(17%)	**36**	(20%)	0.730
	Chronic pain syndrome	**20**	(10%)	**42**	(23%)	<0.001
	Prev angiogram†	**47**	(23%)	**59**	(32%)	0.046
Medications	Aspirin	**185**	(90%)	**161**	(87%)	0.430
	Beta-blocker	**171**	(83%)	**126**	(68%)	0.001
	Calcium channel blocker	**71**	(35%)	**62**	(34%)	0.915
	Nitrates	**83**	(40%)	**83**	(45%)	0.412
	Statin	**187**	(91%)	**153**	(83%)	0.023
	Nicorandil	**29**	(14%)	**32**	(17%)	0.405
	ACE inhibitor or ARB	**108**	(52%)	**83**	(45%)	0.156
Examination/Lab	Systolic blood pressure	**143**	(26)	**138**	(26)	0.031
	Diastolic blood pressure	**75**	(13)	**73**	(13)	0.211
	Pulse	**67**	(11)	**71**	(13)	0.002
	Haemoglobin (g/L)	**14.1**	(1.4)	**13.7**	(1.4)	0.001
	Creatinine (mmol/L)	**82.5**	(18.2)	**80.5**	(42.7)	0.543

		Obstructive CAD (N=206)		INOCA (N=185)		P-value
	Urea	**5.7**	(1.8)	**5.5**	(1.7)	0.212
	Exercise ECG					
	Performed	**131**	(64%)	**118**	(64%)	0.969
	Abnormal	**106**	(81%)	**55**	(47%)	<0.001
	Inconclusive	**19**	(15%)	**46**	(39%)	
	Normal	**6**	(5%)	**17**	(14%)	
Symptoms	Rose-Angina					<0.001
	Definite (Typical)	**174**	(85%)	**118**	(64%)	
	Probable (Atypical)	**32**	(15%)	**67**	(36%)	
	Non-Anginal	**0**	0	**0**	0	
	NYHA I	**49**	(24%)	**25**	(14%)	0.005
	NYHA II	**80**	(39%)	**60**	(32%)	
	NYHA III	**77**	(37%)	**100**	(54%)	
Quality of life	Angina summary score	**53.6**	(19.8)	**51.3**	(18)	0.224
	Angina limitation	**58.3**	(23.3)	**52.5**	(24.4)	0.018
	Angina stability	**50.9**	(28.5)	**45.7**	(24.2)	0.055
	Angina frequency	**60.3**	(27.9)	**60.5**	(23.5)	0.946
	Angina treatment satisfaction	**88.5**	(14.9)	**83.1**	(18.6)	0.001
	Angina quality of life	**42.4**	(22.2)	**40.9**	(21.6)	0.503
	EQ5D – 5L Index Score	**0.65**	(0.23)	**0.60**	(0.28)	0.041
	EQ5D – 5L VAS score	**68**	(21)	**66**	(20)	0.340

TIA – transient ischaemic attack, CVD – cardiovascular disease. ACE – angiotensin converting enzyme inhibitor, ECG – electrocardiogram, NYHA – New York heart association functional classification of dyspnea, EQ5D – European quality of life 5 domain tool for standardized assessment of health outcomes. P-value represents one-way ANOVA for continuous variables or Fisher's exact/Chi squared for categorical variables (adjusted for multiple comparisons). Non parametric data compared using Mann Whitney U test. *10-year risk estimated using validated ASSIGN score calculator † Previous invasive coronary angiogram (at least one previous study).

9. Supplemental Table 3 - Multivariate associates of MVA and VSA in the INOCA population (n=151)

	All MVA (N=109)				All VSA (N=56)			
	**OR**	**95% CI**		**P-value**	**OR**	**95% CI**		**P-value**
Age	1.1	1.0	1.2	0.051	1.1	1.0	1.2	0.032
Female sex	2.7	0.9	7.9	0.063	0.6	0.2	1.7	0.335
Dyspnea								
NYHA I	Ref							
NYHA II	0.8	0.2	3.6	0.795	0.3	0.1	1.2	0.091
NYHA III	0.7	0.2	2.7	0.553	0.3	0.1	1.2	0.095
Smoker								
Never	Ref							
Current	1.6	0.5	5.6	0.427	9.5	2.8	32.7	<0.001
Former	2.2	0.6	8.2	0.255	2.4	0.8	7.1	0.107
ETT								
N/A	Ref							
Abnormal	7.5	1.7	33.0	0.008	0.3	0.1	0.9	0.040
Inconclusive	2.3	0.7	7.3	0.152	0.3	0.1	1.0	0.059
Normal	0.3	0.1	1.4	0.133	0.6	0.1	2.9	0.503
BMI	1.1	1.0	1.1	0.217	1.0	0.9	1.1	0.812
Rose Angina								
Probable (Atypical)	Ref							
Definite (Typical)	2.7	1.1	6.6	0.032	1.2	0.5	3.0	0.663
Diabetic	0.3	0.1	1.9	0.202	4.5	0.9	23.5	0.076
Previous MI	1.7	0.4	6.5	0.460	2.4	0.8	7.3	0.121
FHx CAD	1.5	0.5	4.5	0.458	0.7	0.3	1.7	0.417
Hypertension	1.6	0.6	4.5	0.359	0.6	0.2	1.4	0.230
Dyslipidemia	3.0	1.0	9.0	0.052	0.6	0.2	1.9	0.390
Chronic pain	0.5	0.2	1.7	0.285	1.5	0.5	4.1	0.440
*No CAD**	1.9	0.6	6.1	0.296	0.8	0.3	2.6	0.729
ASSIGN	1.0	0.9	1.0	0.487	0.9	0.9	1.0	0.013

OR: Odds ratio, CAD: coronary artery disease, BMI: body mass index, ETT: exercise treadmill test, NYHA – New York heart association functional classification of dyspnea, * refers to no angiographical evidence of CAD

10. References

1. De Bruyne B, Baudhuin T, Melin JA, Pijls NH, Sys SU, Bol A, Paulus WJ, Heyndrickx GR and Wijns W. Coronary flow reserve calculated from pressure measurements in humans. Validation with positron emission tomography. *Circulation*. 1994;89:1013-22.

2. Fearon WF, Balsam LB, Farouque HM, Caffarelli AD, Robbins RC, Fitzgerald PJ, Yock PG and Yeung AC. Novel index for invasively assessing the coronary microcirculation. *Circulation*. 2003;107:3129-32.

3. Murthy VL, Naya M, Taqueti VR, Foster CR, Gaber M, Hainer J, Dorbala S, Blankstein R, Rimoldi O, Camici PG and Di Carli MF. Effects of sex on coronary microvascular dysfunction and cardiac outcomes. *Circulation*. 2014;129:2518-27.

4. Hashimoto S, Kobayashi A. Clinical pharmacokinetics and pharmacodynamics of glyceryl trinitrate and its metabolites. Clin Pharmacokinet 2003;42(3):205-21.

5. Reriani M, Raichlin E, Prasad A, Mathew V, Pumper GM, Nelson RE, Lennon R, Rihal C, Lerman LO and Lerman A. Long-term administration of endothelin receptor antagonist improves coronary endothelial function in patients with early atherosclerosis. *Circulation*. 2010;122:958-66.

6. Suwaidi JA, Hamasaki S, Higano ST, Nishimura RA, Holmes DR, Jr. and Lerman A. Long-term follow-up of patients with mild coronary artery disease and endothelial dysfunction. *Circulation*. 2000;101:948-54.

7. Rose G, McCartney P, Reid DD. Self-administration of a questionnaire on chest pain and intermittent claudication. Br J Prev Soc Med. 1977 Mar;31(1):42–48.

8. Spertus JA, Winder JA, Dewhurst TA, Deyo RA, Prodzinski J, McDonnell M, et al. Development and evaluation of the Seattle Angina questionnaire: A new functional status measure for coronary artery disease. J Am Coll Cardiol 1995;25(2):333-341.

9. Chan PS, Jones PG, Arnold SA, Spertus JA. Development and validation of a short version of the Seattle angina questionnaire. Circ Cardiovasc Qual Outcomes 2014;7(5):640-7.

10. EuroQoL Group (1990) EuroQoL: a new facility for the measurement of heath-related quality of life. Health Policy16:199–208.
